# Antigen binding triggers long-range conformational changes in monoclonal antibodies

**DOI:** 10.3389/fimmu.2025.1680199

**Published:** 2026-01-08

**Authors:** Davide Bianchi, Simona Saporiti, Wolf Palinsky, Omar Ben Mariem, Mara Rossi, Ivano Eberini, Fabio Centola

**Affiliations:** 1 Dipartimento di Scienze Farmacologiche e Biomolecolari, Università degli Studi di Milano, Milan, Italy; 2 Analytical Excellence and Program Management, Merck Serono S.p.A., Rome, Italy; 3 Global Chemistry, Manufacturing, and Controls (CMC) Development, Merck Healthcare, Corsier-sur-Vevey, Switzerland; 4 Dipartimento di Scienze Farmacologiche e Biomolecolari & Data Science Research Center (DSRC), Università degli Studi di Milano, Milan, Italy

**Keywords:** accelerated molecular dynamics, ADCC, FcγRIIIa, monoclonal antibodies, N-glycosylation

## Abstract

**Introduction:**

Antigen binding in monoclonal antibodies (mAbs) is traditionally associated with target recognition, but emerging evidence suggests it may also modulate antibody conformation and effector functions. Understanding how antigen engagement impacts the structural organization of therapeutic antibodies and their interaction with immune receptors is essential for optimizing antibody-based therapies. In this study, we investigated, *via* computational approaches, how antigen engagement alters the structural organization of therapeutic IgG1s and influences their ability to interact with FcγRIIIa.

**Methods:**

Accelerated molecular dynamics simulations were performed to investigate the structural and dynamic consequences of antigen binding in two therapeutic mAbs, adalimumab and avelumab. These antibodies were analyzed in different glycosylation states to capture the influence of post translational modifications.

**Results:**

**T**he results revealed consistent long-range dynamic correlations between antigen-binding regions and distant domains within the Fc, suggesting an allosteric communication network involving the entire antibody structure and mediated by the antigen binding. Antigen engagement was found to increase the exposure of Fc residues critical for immune receptor recognition, an effect modulated by glycosylation and light chain isotype.

**Conclusion:**

These findings suggest that antigen engagement initiates a cascade of coordinated motions that reshape the mAb architecture and regulate its interaction with the immune receptors, offering new insights for the design of functionally optimized therapeutic antibodies.

## 1 Introduction

Therapeutic monoclonal antibodies (mAbs) are immunoglobulin G (IgG) molecules clinically validated for the treatment of a broad range of diseases as a result of their ability to bind target antigens with high specificity and long-term affinity. Structurally, they consist of two identical heavy chains (HCs) and two identical light chains (LCs), forming a Y-shaped heterodimer with an approximate molecular weight of 150 kDa. These polypeptide chains are stabilized by inter- and intra-chain disulfide bonds, maintaining the antibody structural integrity (1, 2). Each chain comprises both variable and constant domains. The antigen-binding portion of the antibody is known as Fab region, which includes the variable fragment (Fv), composed of the variable domains of the heavy (VH) and light (VL) chains, and the constant domains (CH1 and CL) (3). The Fc region, formed by the remaining constant domains of the HCs (CH2 and CH3), is on the other hand responsible for mediating immune effector functions. mAbs exhibit two main functions: specific recognition of target antigens via non-covalent interactions and, consequently, the activation of immune responses (4). The recognition occurs via the Fv region, where complementarity-determining regions (CDRs) form the antigen-binding site, also known as the paratope, which specifically binds to a defined region of the antigen called epitope. The CDRs are hypervariable loops whose sequence and structure determine the specificity and affinity for antigen binding. Each antibody contains three CDRs on the LC (L1–L3) and three on the HC (H1–H3) (5). Albeit all mAbs exhibit their function by targeting antigens, their downstream mechanism of action is further modulated by the engagement of the Fc domain with Fcγ receptors (FcγRs) that is critical for initiating immune system activation and triggering effector functions such as antibody-dependent cellular cytotoxicity (ADCC) (6). To prevent inappropriate immune responses, FcγR stimulation is tightly regulated and typically occurs only when antibodies are bound to their specific antigens, an event that facilitates receptor clustering on the cell surface (7–9). Among the FcγRs, FcγRIIIa plays a central ADCC-activating role, and therefore is significantly relevant from a therapeutic perspective (10). It is well recognized that the activation of mAbs effector functions is modulated by structural attributes determined by post-translational modifications (PTMs), with glycosylation playing a key role (11). Variations in glycosylation patterns constitute a major source of heterogeneity among mAbs, influencing both their biological activity and therapeutic efficacy (12). N-linked glycosylation at the conserved Asn297 (according to Eu numbering (13)) in the Fc region is particularly critical because it is essential for maintaining the structural conformation of the Fc domain and for enabling interactions with FcγRs, directly impacting effector functions such as ADCC (14–16). Accordingly, X-ray crystallography studies of different Fc domains with distinct glycan truncations have shown that the aglycosylation at Asn297 leads to progressively more closed Fc conformations, bringing the CH2 domains closer together compared to the fully glycosylated, open Fc structure (17). Furthermore, the specific glycosylation pattern at Asn297 influences the overall structure of the Fc domain as well as the pharmacokinetics (PK) and pharmacodynamics (PD) of mAbs (18). In this context, core fucosylation has been previously shown to consistently reduce the binding affinity of IgG1 to FcγRIIIa, thus reducing the ADCC response (19–24). While the Fc domain has traditionally been considered functionally and structurally distinct from the variable regions, emerging evidence suggests a more integrated model of antibody behavior. It has been reported that the constant regions of mAbs are not only responsible for the effector functions but may also influence the conformational behavior of the variable regions and, therefore, the affinity for the antigen (25).

Despite extensive research in this field, the precise nature and extent of the structural rearrangements occurring in the Fc region upon antigen binding remain poorly defined. In this context, our study aims to deepen the understanding of antigen-induced conformational effects by investigating the structural changes that mAbs undergo upon the interaction. The final scope of the study is to evaluate if a specific path from antigen binding regions to Fc domain exists in terms of short- and long-range structural correlations that can modulate the antibody function.

To this purpose, we employed accelerated molecular dynamics (aMD) simulations on two commercial mAbs, adalimumab and avelumab, analyzed in both fucosylated and afucosylated states, G0 and G0F, respectively. As detailed in our previously published works, adalimumab and avelumab were used as case studies since they are well described biopolymers that also differ in the LC composition (with a κ isotype for adalimumab and λ isotype for avelumab), another variable that has been shown to influence the conformational variability of mAbs (16, 26). Both adalimumab and avelumab can elicit ADCC responses upon interaction with their antigens on the target cells, respectively transmembrane TNF-α for adalimumab (27), and PD-L1 for avelumab (28). Given these features, and since they do not present any modification in the hinge or in the Fc regions, these antibodies can be considered good models for studying the impact of antigen engagement on FcγRIIIa interaction.

## 2 Materials and methods

### 2.1 Homology modeling

Three-dimensional (3D) models of adalimumab and avelumab, each in complex with their respective antigens, were generated using a chimeric homology modeling approach via the Homology model tool of the MOE 2022.02 software (29). The only fully crystallized human IgG1 structure (PDB ID: 1HZH) (30) was employed as a template for the antibody backbone and for modeling the hinge and Fc portions. To correctly orient the domains in space, X-ray structures of adalimumab and avelumab Fab domains (PDB IDs: 3WD5 and 5GRJ, respectively) (31, 32), were superposed to 1HZH structure. Subsequently, the crystallized Fab domain of each mAb was used as a template to model the corresponding variable regions, while the Fc and hinge regions were modeled based on the 1HZH structure. Prior to the modeling procedure, all the templates were prepared using the Structure preparation tool included in MOE to solve any crystallographic issues and remove solvent molecules. Protonation states and hydrogen placement were refined using the Protonate 3D tool, which assigns appropriate ionization states and adds missing hydrogen atoms.

In 3WD5, a loop consisting of five amino acids between Ser136 and Gly152 in the HC was built using the Build Loop option of the Structure preparation, which is optimized for generating loops shorter than six residues. The N- and C-termini of TNF-α were capped with acetyl (ACE) and N-methyl amide (NME) groups, respectively. Additionally, Gly212, Glu213, and Cys214 residues in LC of 3WD5 were modeled using the template override option with 1HZH as reference. For 5GRJ, the residues Ser119 and Ser120 in the HC, as well as Gln1 and Leu110 in the LC were modeled based on the corresponding residues in 1HZH by using the template override option. Considering the dimension of PD-L1, only the portion of the antigen identified by Liu et al. as the epitope (from residue 18 to 134) was included in the model (31). The resulting mAbs::antigen complexes were then N-glycosylated at the conserved Asn297 residue. As the 1HZH template contains G0F glycans, these were transferred *via* structural superposition and linked to the conserved Asn297 sites in both antibodies to obtain the fucosylated models as described in our previous studies (16, 33). Specifically, the glycans were covalently linked to the Fc via bond between the amide nitrogen of Asn297 and the anomeric carbon of the first N-acetylglucosamine residue. Local energy minimization was then applied to Asn297 and the attached glycans until a root mean square (RMS) gradient of 0.01 kcal/mol/Å^2^ was reached. To obtain the afucosylated (G0) variants, fucose units were removed from the glycan chains in the previously generated G0F models, followed by local minimization of Asn297 and the modified glycans using the same convergence threshold (0.01 kcal/mol/ Å^2^).

Finally, the generated complexes in the different glycosylation states were subjected to global energy minimization toward a RMS gradient of 0.001 kcal/mol/Å^2^ to solve steric clashes and prepare the structures for further analyses.

### 2.2 Molecular dynamics simulations

The mAb::antigen complexes were subjected to short 50 ns long classical molecular dynamics (MD) simulations using AMBER22 (34) software and the CHARMM36 forcefield (35) to obtain the average data needed for the accelerated MD. Specifically, each complex was placed in a cubic periodic box with a distance of 15 Å from the edges and the systems were solvated using TIP3P water model and NaCl 0.15 M to neutralize the charge. The box dimension for each system is: 218 Å^3^ (G0 adalimumab), 223 Å^3^ (G0F adalimumab), and 201.6 Å^3^ (G0 and G0F avelumab). The minimization phase was performed for 5000 cycles, consisting of 2500 initial steps of steepest descent followed by conjugated gradient minimization. During this phase, to preserve the initial conformation of key regions, positional restraints were applied to the protein backbone and to the N-linked glycans. Additionally, dihedral restraints were applied to sugars torsion angles. The particle mesh Ewald (PME) method (36) was used to handle long-range interactions with a 12 Å cutoff and the SHAKE algorithm (37) was applied to constraint hydrogen atoms vibration by fixing all bonds involving hydrogens. Each equilibrated complex was then subjected to 1 μs long aMD simulations. The production phase was carried out in NPT ensemble (T = 300 K, P = 1 bar) using Langevin dynamics and Berendsen barostat with a time step set to 0.002 ps. The coordinates and energies were saved every 50,000 steps (100 ps) for subsequent analyses. Boost potentials were applied to both the dihedral and total potential energy terms *(iamd=3)* using the parameters EthreshD and alphaD for the dihedral component, and EthreshP and alphaP for the total potential energy (38). EthreshD, EthreshP, alphaD and alphaP were calculated according to the equations reported in our previous work (16). The values of the parameters used as input for the aMD simulations are reported in **Supplementary Table S1** in Supplementary materials.

### 2.3 Analysis of aMD trajectories

Considering the addition of a boost in the aMD simulations, a reweighting procedure was necessary to recover the original free energy landscape of the studied molecules. Here, the method used by Miao et al. (39) was applied using Maclaurin expansion to the 10^th^ order to approximate the free energy surface (FES) of the system as a function of θ angles. These angles, as thoroughly described in our previous work (33), describe the motion of the Fab domains with respect to the Fc region and represent a suitable descriptor able to study the global conformational behavior of mAbs. The correlation matrices were obtained by calculating the covariance matrices of C-α atoms fluctuation along the aMD simulations trajectories. Solvent accessible surface area (SASA) was computed using the surf tool of CPPTRAJ (34), that calculates the surface area of a solute molecule that is accessible to solvent molecules using the LCPO algorithm (40). SASA was calculated for each of the key residues for FcγRIIIa interaction identified by Shields et al. via mutagenesis studies (41). Principal component analysis (PCA) was computed on the aMD trajectories to identify and characterize the dominant collective motions of the system. The covariance analysis tool of GROMACS (42) was used. PCA was applied to the positional fluctuations of backbone atoms after structural alignment, and the major modes of motion were extracted by diagonalizing the covariance matrix of atomic fluctuations. The distance between the CH2 domains was computed as distance between the centers of mass of the domains using the python library MDAnalysis (43). For each frame of the trajectory, the center of mass (COM) of each CH2 domain was calculated using atomic masses, then the distance between the two centers of mass was determined. The distance was used as a reaction coordinate to compute the potential of mean force (PMF) via reweighting of the aMD data. The hydration free energies were computed using the Solvent Analysis tool included in MOE to estimate the solvent exposure and hydration thermodynamics of residues critical for FcγRIIIa interaction. The solute mode of the 3D-RISM was used, and cutoff distance of 5 Å with respect to the selected atoms was chosen. Only water densities associated with negative hydration free energy (dG) values were considered, as they represent thermodynamically favorable solvation sites. The contacts between the CH1 and CL domains were computed using the nativecontacts tool of CPPTRAJ applying a threshold distance of 4 Å. The residue-specific interaction interface between the CH1 and CL domains was identified through the Protein contacts tool available in MOE, that calculates the possible contacts between two sets of atoms (in this context CH1 and CL residues), computes the associated interaction energies and allows the generation of the interaction surfaces. The interaction energies between these residue pairs were computed over the trajectories of the energetic minima to obtain more statistically reliable results. This calculation was performed using the energy command of CPPTRAJ provided by AmberTools, that computes different kind of energy terms. The electrostatic component was taken into consideration, and the mean was computed for each residue. All data referred to the unbound states of the mAbs originate from already published aMD simulations trajectories (16) that were reanalyzed.

## 3 Results

### 3.1 Identification of energetic minima

**Figure 1:**
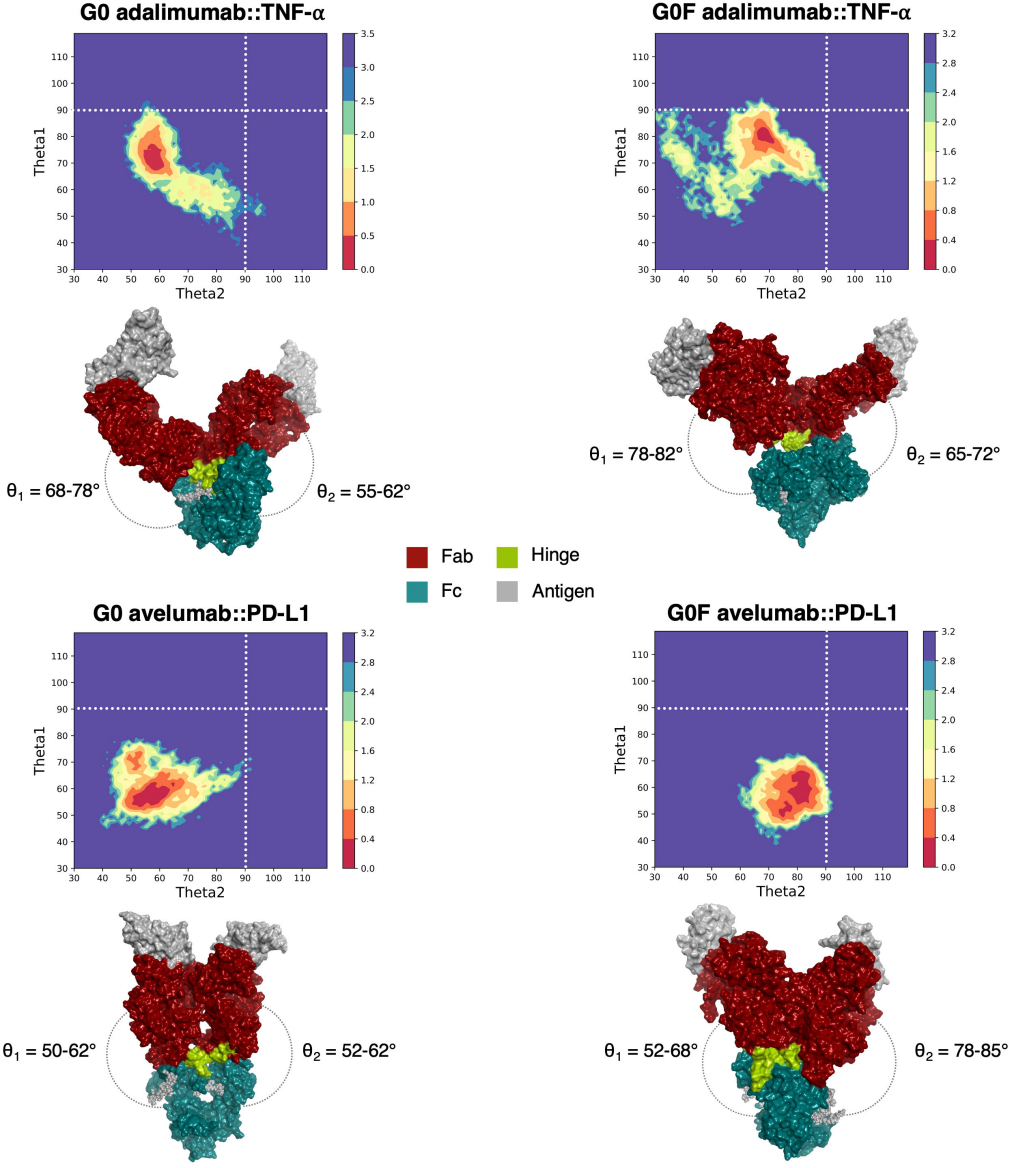
Free energy profiles for the simulations of the four mAb::antigen complexes. Free energy profiles are plotted as a function of the θ angles. The colorbar represents the potential of mean force (PMF) in kcal/mol. Corresponding representative structures of each energetic minimum are shown in molecular surfaces to highlight the conformational features of each system.

To characterize the conformational space explored by mAbs upon antigen binding, free energy profiles were generated as a function of the θ angles, which define the relative orientation of the Fab domains with respect to the Fc region. As previously established (33), these angles represent a reliable descriptor of the global conformational behavior of IgGs. The resulting PMF surfaces, shown in **Figure 1**, allowed the identification of the lowest energy conformational states (defined as PMF values < 0.5 kcal/mol) for each mAb::antigen complex. Although each system reached an energetic minimum at distinct θ values, all of them converged toward conformational states consistent with a canonical Y-shaped structure (θ < 90°). The distribution of θ angles can be also used to discriminate between distinct global conformations: values below 90° are associated with Y-like states, while values higher than 90° reflect more closed, T-like arrangements of the Fab domains that gets in proximity to the Fc. Representative structures of the mAbs sampled at the energy minima are shown in **Figure 1**, highlighting the characteristic Y-shaped arrangement of the mAb::antigen complexes. Specifically, in G0 adalimumab system, the energetic minimum was located around θ_1_ = 68-78° and θ_2_ = 55-62°; for G0F adalimumab, the minimum shifted to θ_1_ = 78-82° and θ_2_ = 65-72°. In G0 avelumab system, the minimum was observed between θ_1_ = 50-62° and θ_2_ = 52-62°, whereas in G0F avelumab it was identified around θ_1_ = 52-68° and θ_2_ = 78-85°. Interestingly, the low-energy conformations identified upon antigen binding notably differ from those observed in our previous simulations of the unbound antibodies (16), revealing that upon antigen binding all mAbs assume very similar shapes. A comparison between the different conditions is reported in **Supplementary Figures S1**, **Supplementary Figures S2**, showing that, in the unbound state, only G0 adalimumab exhibited a Y-shaped conformation, characterized by θ values < 90°, resulting in open Fab orientations. In contrast, all other unbound systems adopted more compact, T-shaped conformations, with at least one θ angle > 90°.

### 3.2 Covariance analysis

**Figure 2:**
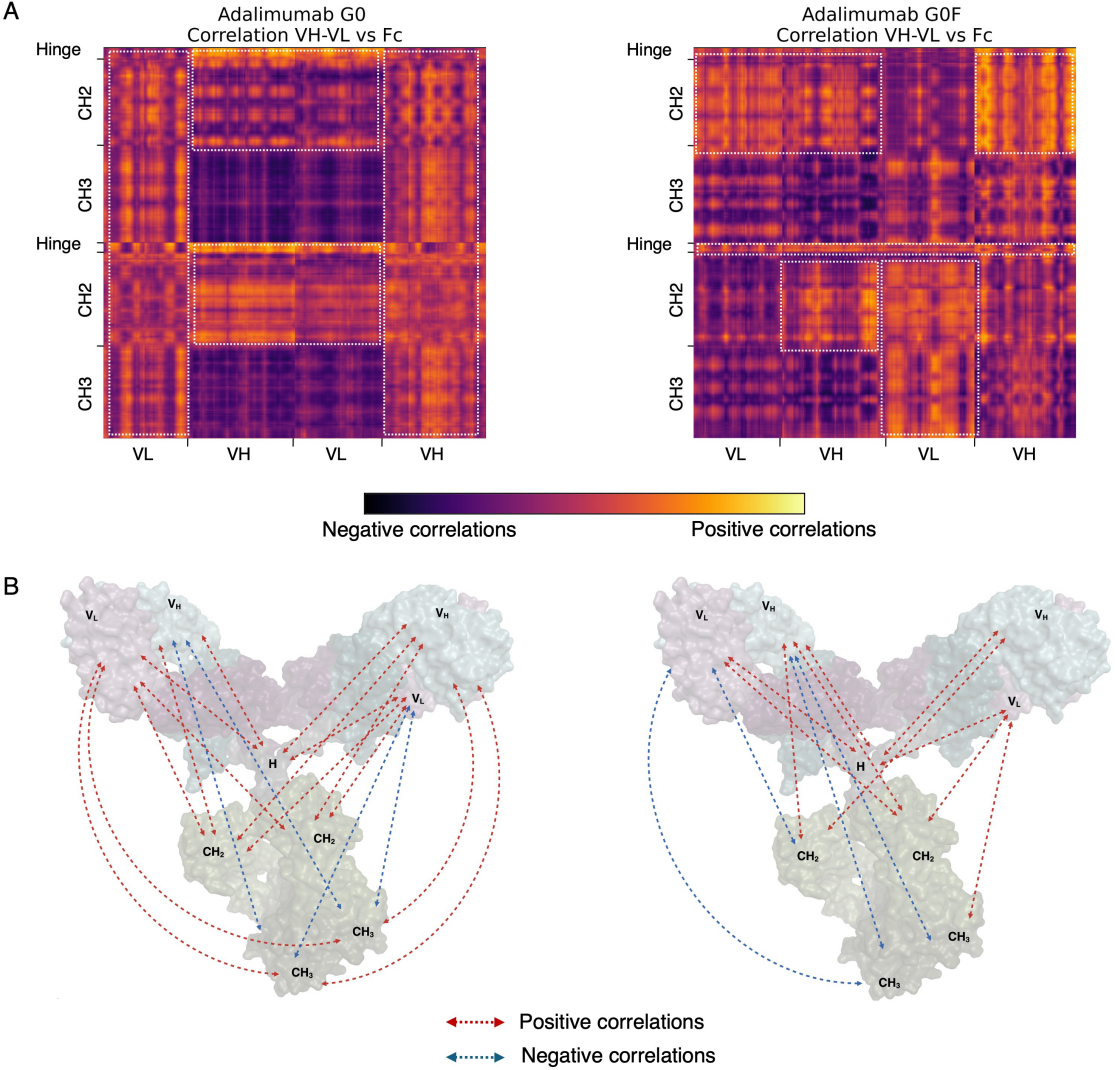
Long-range correlations derived from the covariance analysis computed for adalimumab::TNF-α complexes. **(A)** Correlation matrices between the variable regions of the Fab (VH, VL) and the Fc and hinge region. Relevant correlations are delimited by white boxes. **(B)** Schematic representation of the observed correlations. Arrows indicate correlated motions between the variable regions of the Fab domains and the Fc and hinge region: red arrows represent positive correlations, while blue arrows denote negative correlations. H: hinge.

**Figure 3:**
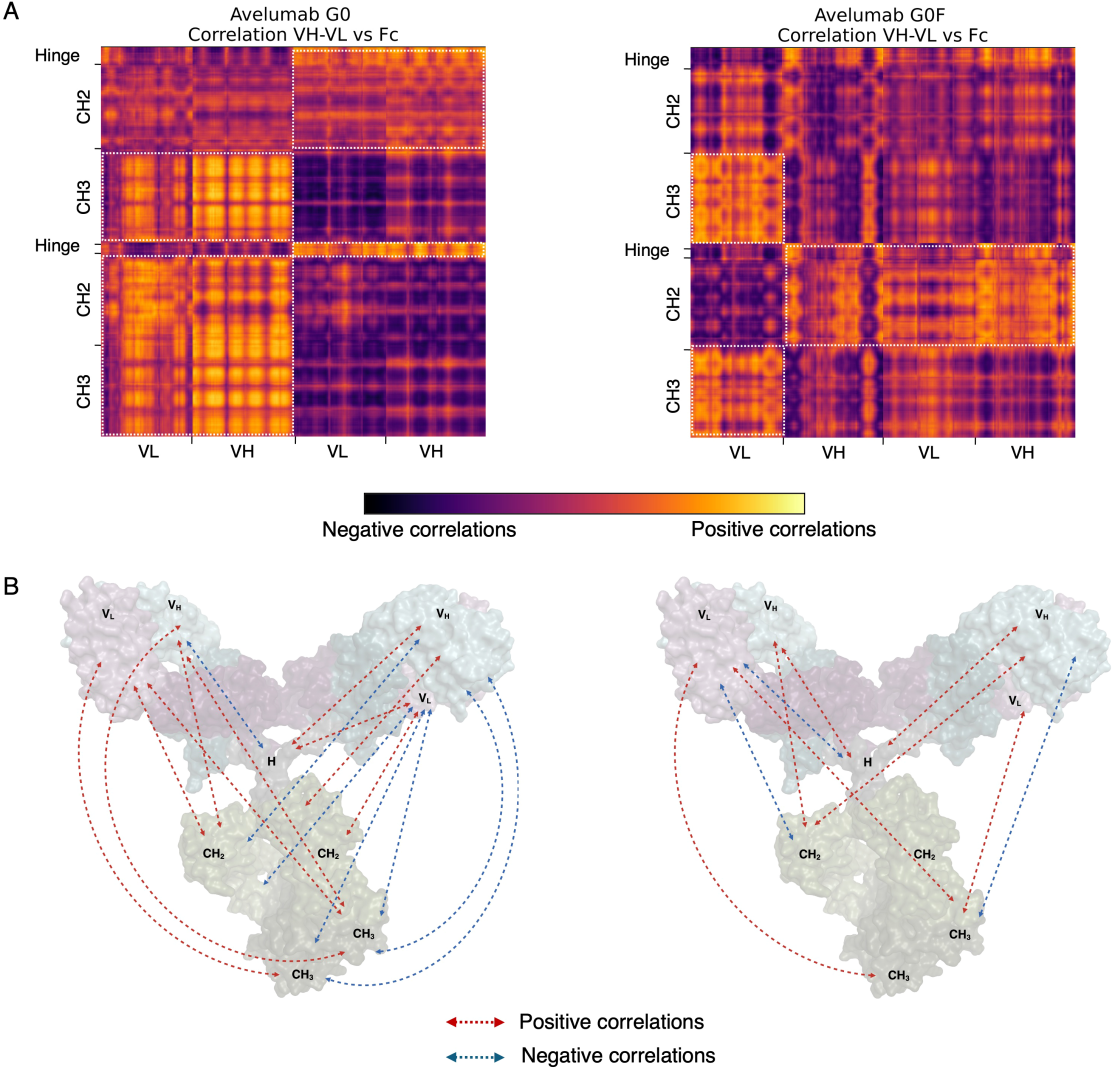
Long-range correlations derived from the covariance analysis computed for avelumab::PD-L1 complexes. **(A)** Correlation matrices between the variable regions of the Fab (VH, VL) and the Fc and hinge region. Relevant correlations are delimited by white boxes. **(B)** Schematic representation of the observed correlations. Arrows indicate correlated motions between the variable regions of the Fab domains and the Fc and hinge region: red arrows represent positive correlations, while blue arrows denote negative correlations. H: hinge.

To characterize the internal dynamics of the mAbs upon antigen binding, a covariance analysis based on C-α atom fluctuations of the antibody was performed. This method highlights patterns of correlated and anti-correlated motions, revealing how different regions of the antibody exhibit correlated or opposing dynamic behaviors. Across all systems, we observed strong, long-range positive correlations between the variable domains of the Fab (VH and VL) and distant regions of the mAbs such as the hinge and Fc, including both CH2 and CH3 domains. These correlations, represented in **Figure 2** (adalimumab) and **Figure 3** (avelumab), are particularly interesting given the considerable distance between these domains in the antibody structure. Although correlated motions usually occur between regions that are structurally close or directly connected, the strong interdomain correlations observed indicate a high level of dynamic coordination within the mAb. Interestingly, while this trend was consistent across all mAbs, differences in the strength and extent of these correlations were noted depending on N-glycosylation. Antibodies with afucosylated glycans generally exhibited a higher number of strong, positive interdomain correlations compared to their fucosylated counterparts, which instead displayed fewer and sometimes even negative correlations. Additional correlation patterns were detected between the constant regions of the Fabs (CH1 and CL) and the hinge or Fc domains, further supporting the idea that the coordination between the Fabs and Fc is not limited to the Fv but involves a broader dynamic network. These extended correlation networks are reported in **Supplementary Figure S3**.

### 3.3 Antigen binding affects the conformational behavior of the Fc

**Figure 4:**
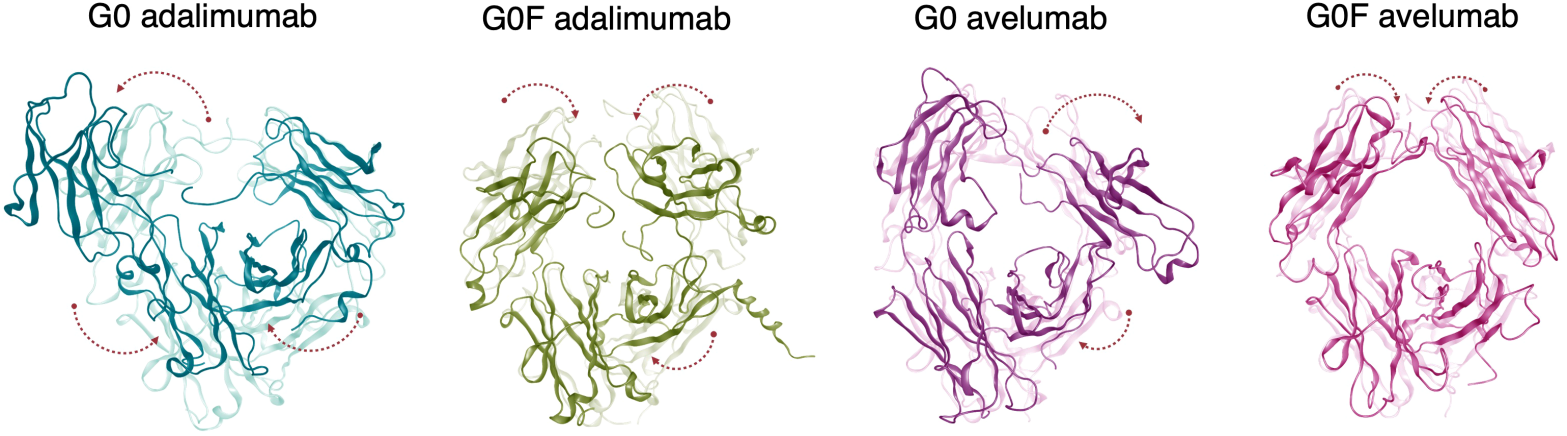
Principal motions of the Fc domain derived from PCA. The dominant motions of the Fc region are represented based on principal component analysis. The background structure corresponds to the initial conformation at the beginning of the simulation, while the foreground structure shows the Fc conformation at the energy minimum along the principal components. Arrows indicate the direction of the CH2 and CH3 domains movements, highlighting the collective motions captured by the first principal mode.

**Figure 5:**
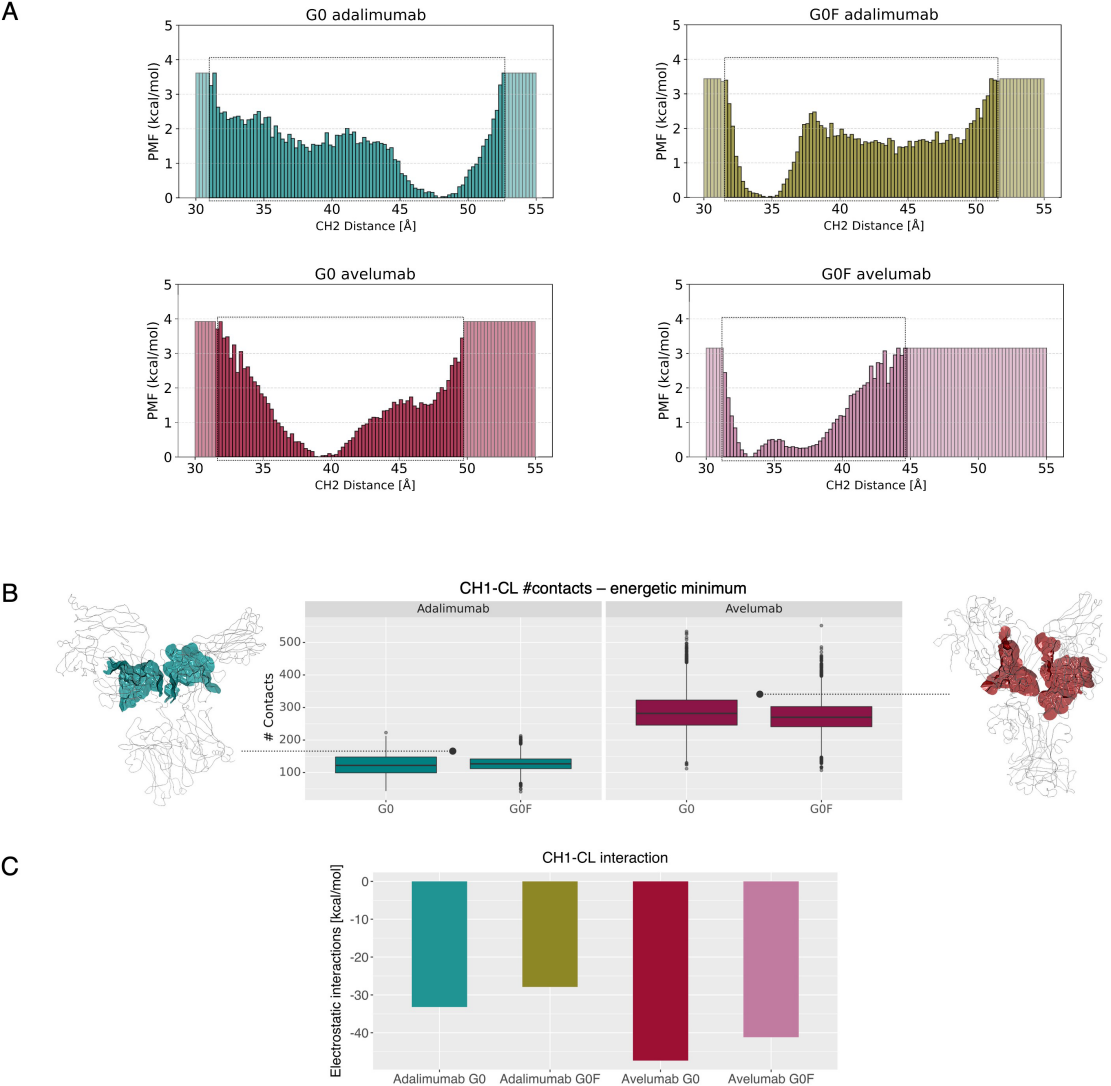
Analysis of Fc conformational behavior and Fab stabilization across glycosylation mAb::antigen complexes. **(A)** Free energy profiles along CH2 distance. For each system the PMF is reported over a fixed distance range to allow direct comparison across the mAbs. The actual distance ranges sampled during the simulations are highlighted with boxes within each plot, indicating the span of conformational space explored by each system. **(B)** Number of contacts between the CH1 and CL domains throughout the trajectories of the minima. Representing Fab conformations are reported with the interaction surface identified by the Protein contacts analysis. **(C)** Barplots showing the mean electrostatic interaction energies (kcal/mol) of key CH1-CL residue pairs identified in the energy minima using the Protein Contacts tool.

To gain further insight into the conformational dynamics of the antibodies, we performed a PCA on the aMD trajectories. This analysis allowed the isolation of the dominant collective motions exhibited by the systems during the simulations. Specifically, we focused on the first principal component (PC1), which describes the largest variation in the Fc dynamics across all systems. As shown in **Supplementary Figure S4**, the first two PCs together account for most of the conformational variability observed. The motions associated with PC1s were examined by projecting each trajectory along this component and mapping the corresponding directional vectors onto representative mAb structures. In **Figure 4**, a graphical representation of the movements occurring in the Fc is reported with a comparison to the first frame of each simulation to highlight the nature of the structural rearrangements leading to the conformations observed in the energetic minima. These changes are predominant in the upper-CH2 domains, suggesting a major impact of antigen binding on these portions of the region. To quantify these conformational changes, the distance between the COMs of the CH2 domains was computed along the aMD trajectories. This descriptor was chosen to quantify the degree of Fc opening and closure over time and it was used as a reaction coordinate to compute the PMF via reweighting of the aMD data (**Figure 5A**). These results reveal distinct energetic behaviors across mAbs depending on both glycosylation states and LC isotype. Adalimumab explores a broader range of CH2 distances, with both G0 and G0F variants sampling values between approx. 31 Å and 53 Å. However, the location of the energy minima substantially differs between the states: G0 variant exhibits a minimum at 48 Å, corresponding to an open Fc conformation, while the G0F form displays a minimum at 35 Å, reflecting a more compact arrangement induced by fucosylation. In contrast, avelumab samples a narrower range of CH2 distances, suggesting overall reduced Fc flexibility. The G0 variant reaches a minimum at 40 Å, while the G0F form shifts to a more closed state, with a minimum at 34 Å. These results suggest that adalimumab presents greater conformational flexibility in the Fc, whereas avelumab shows a more constrained dynamic profile of this domain. To understand the structural determinants underlying these differences, we hypothesized that interactions between the CH1 and CL domains in the Fab might influence the downstream dynamics of the Fc. The number of interdomain contacts between CH1 and CL along the frames of the energetic minima was computed. As shown in **Figure 5B**, avelumab consistently exhibits a higher number of CH1-CL contacts compared to adalimumab, regardless of glycosylation. This suggests a tighter and more stabilized Fab arrangement in avelumab due to the λ-LC isotype, which could act as a constraint on hinge flexibility limiting Fc motion. To further explore this hypothesis, we performed an analysis of the interaction interfaces between CH1 and CL in the minimum-energy structures. The interface surfaces between CH1 and CL domains were mapped and reported in **Figure 5B**, and key residue pairs contributing to domain stabilization were identified based on their interaction energy, that was then computed along the minimum-energy frames. Considering that most of the identified interactions are H-bonds, only the electrostatic component of the energy was considered for the analysis and the mean electrostatic energy of all the contacts of each system is reported in **Figure 5C**. Avelumab consistently shows more negative interaction energies compared to adalimumab in both glycosylation states, indicating a more stable CH1-CL interface in presence of the λ-LC isotype.

### 3.4 Effect of antigen interaction on the exposure of key residues for FcγRIIIa interaction

**Figure 6:**
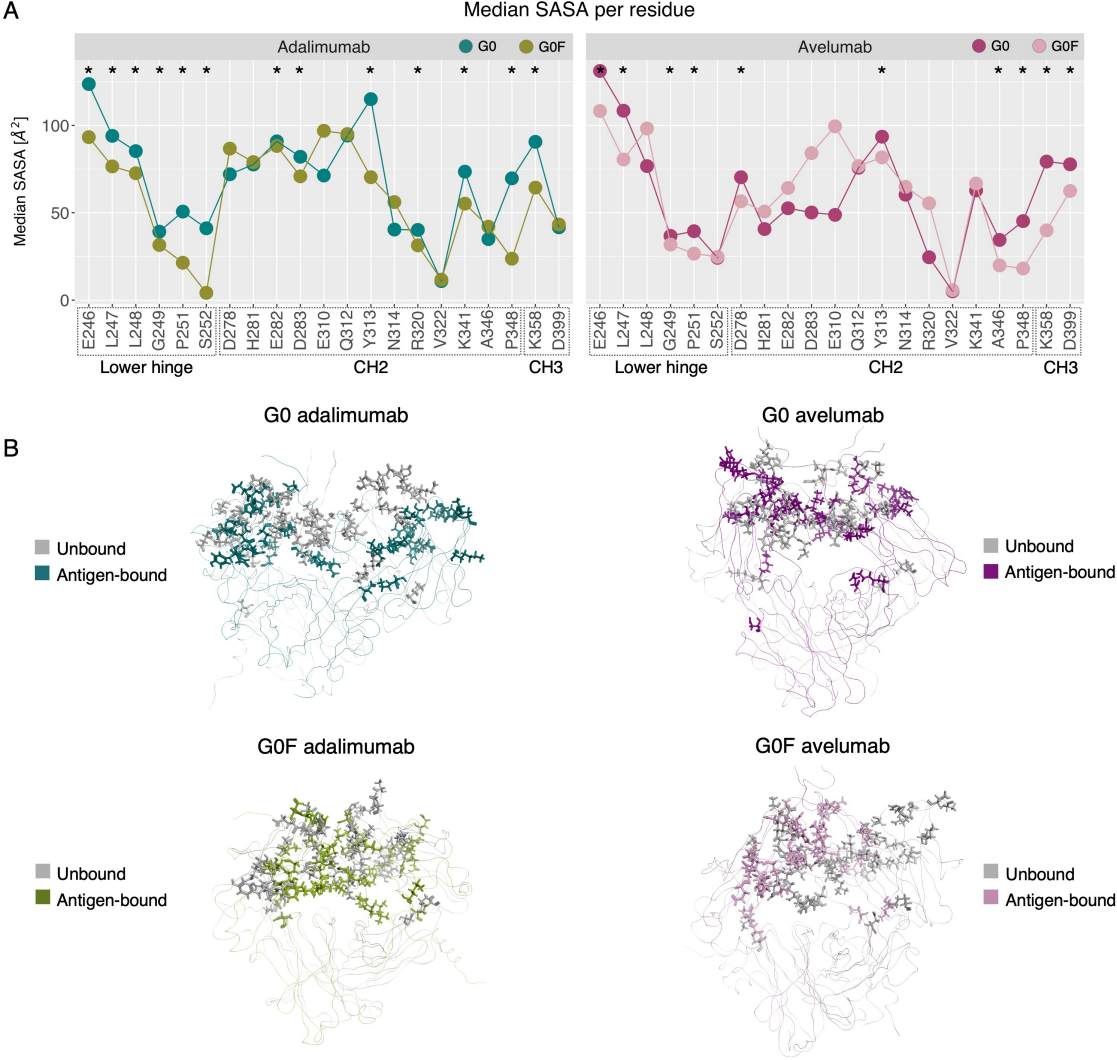
Effect of antigen binding on the solvent exposure of FcγRIIIa-interacting residues. **(A)** Average SASA values for key FcγRIIIa-binding residues across the mAb::antigen complexes, computed in the minimum-energy conformations. **(B)** Structural superposition of the Fc domains in antigen-bound (colored) and unbound (gray) states, highlighting conformational changes upon antigen engagement.

**Figure 7:**
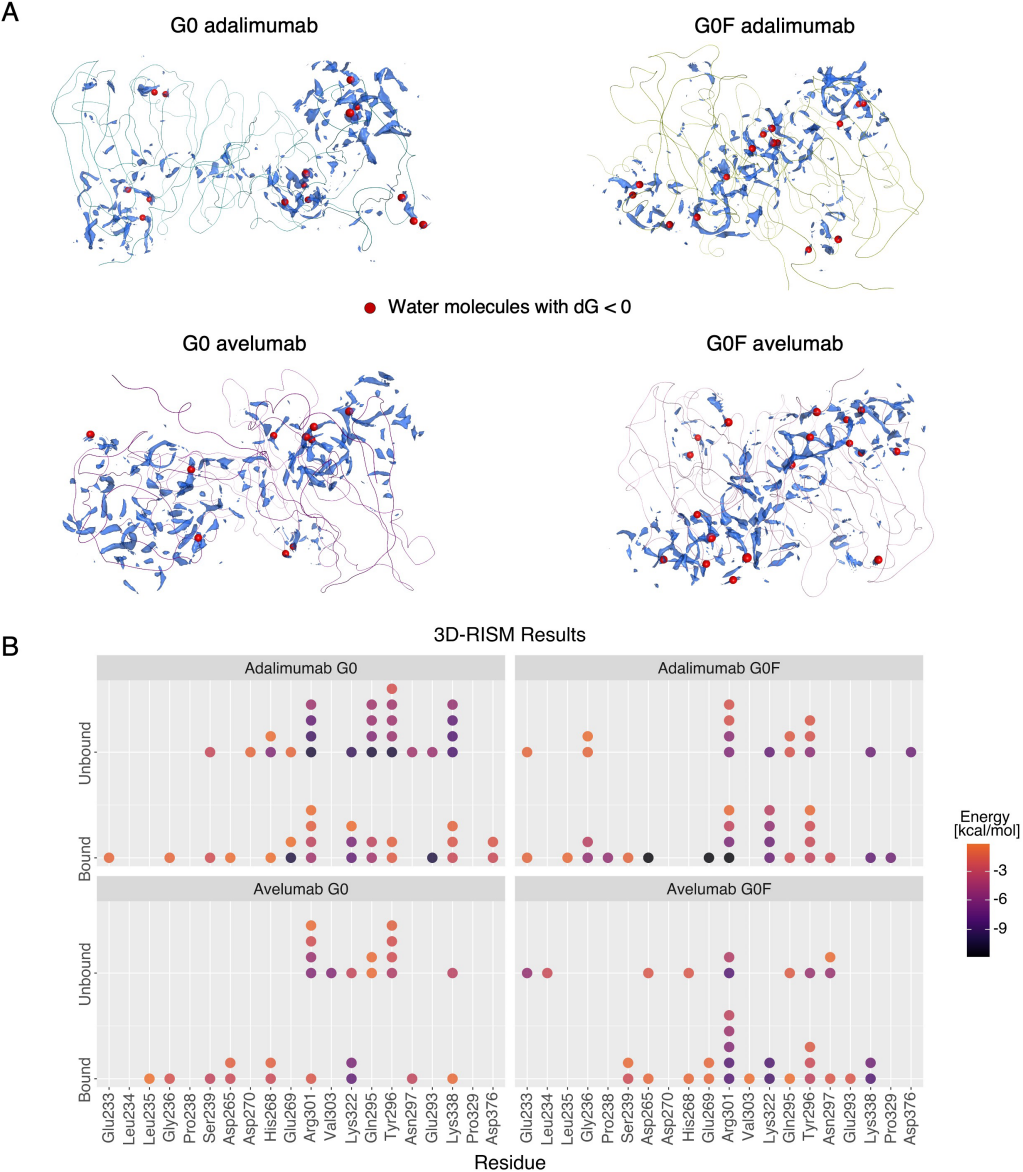
Thermodynamic effect of antigen binding on solvation of FcγRIIIa-interacting residues. **(A)** Representative 3D-RISM hydration maps showing the most thermodynamically favorable water interaction sites (red dots) around key interacting residues for each system in the antigen-bound state. **(B)** Dotplot representing the hydration free energies of key Fc residues across the complexes in both antigen-bound and unbound states. Each point corresponds to a hydration site on a specific residue, the color scale reflects the hydration energy (kcal/mol), with darker shades indicating more favorable water::residue interactions.

To evaluate how antigen binding modulates the accessibility of Fc residues involved in immune receptor recognition, we calculated the solvent-accessible surface area (SASA) for a set of key residues previously identified by Shields et al. (41) as critical for FcγRIIIa binding. These residues, primarily located in the lower hinge and surrounding regions of the CH2 domains, are known to significantly contribute to receptor affinity. **Figure 6A** reports the average SASA values for each residue within the energy minima of the four systems, allowing for a direct comparison between the G0 and G0F states. In adalimumab, a clear trend emerges, by which most of the studied residues are more exposed in the G0 variant than in G0F, consistent with a more open Fc conformation in the afucosylated state. This aligns with the CH2 distances observed in the same energy minimum, reinforcing the notion that fucosylation promotes more closed Fc conformations and highlighting its possible contribution to reduce the accessibility of receptor-binding surfaces. In contrast, avelumab displays a more balanced SASA distribution between G0 and G0F forms. This reduced difference between the glycosylation states is in line with the trend previously observed in CH2 distances, where the gap between G0 and G0F is less pronounced than in adalimumab. To further understand how antigen binding affects these receptor-interacting residues, the antigen-bound structures were compared with their unbound counterparts previously characterized in solution (16). In **Figure 6B**, the structural superposition of the Fc domains revealed that, upon antigen engagement, in G0 mAbs key residues for receptor binding can rearrange toward a more solvent-exposed configuration. In contrast, G0F antibodies show limited structural rearrangement in the same regions, maintaining a more buried and compact Fc conformation. To characterize the exposure of FcγRIIIa-interacting residues to a thermodynamical extent and to explore how it is affected by antigen engagement, a solvent analysis via the 3D-RISM was performed for the minimum-energy conformations of each system, in both the bound and unbound states. The analysis was specifically focused on the negative hydration free energy values of key receptor-interacting residues, which are indicative of the thermodynamic stability of local water::residue interactions. **Figure 7A** shows the structural localization of the key solvation sites, while **Figure 7B** reports their associated hydration energy for each system. Across complexes, the antigen-bound state is generally associated with more negative hydration free energies at key Fc residues compared to the unbound state, indicating increased solvent exposure upon antigen engagement. This trend is especially marked in G0F adalimumab, where Glu269, Arg301, and Asp265 show a dramatic decrease in solvation energy values, from moderately negative values in the unbound form to even lower values up to -11.98 kcal/mol in the bound state. This shift indicates that antigen binding promotes pronounced conformational rearrangements that compensate for the structurally compact nature of the Fc induced by core fucosylation. In contrast, G0 adalimumab displays relatively stable hydration energies across both states. Key residues like Gln295, Lys322, and Arg301 in fact already exhibit highly negative hydration energies in the unbound form, suggesting that these regions are solvent-accessible even prior to antigen engagement. On the other hand, in avelumab, both G0 and G0F states show a moderate but consistent shift toward more negative hydration energies in the bound state. These findings align with the smaller conformational changes observed in SASA and CH2 domain distances and confirm that, even depending on glycosylation, Fc region in avelumab is less flexible than adalimumab.

## 4 Discussion

The present study aims to explore the conformational impact of antigen binding on the structure and function of therapeutic mAbs, with a specific focus on the Fc region and its interaction with the immune receptor FcγRIIIa. Using adalimumab and avelumab as model systems and comparing their G0 and G0F glycosylation states, we employed aMD simulations to investigate their dynamic behavior in complex with the cognate antigens, TNF-α and PD-L1, respectively. These two biotherapeutics are representative of different LC isotypes (κ for adalimumab, λ for avelumab) and different FcγRIIIa interaction profile, allowing the investigation of how antigen binding affects mAb architecture and potentially modulates the effector functions. The conformational flexibility of the Fc and the extent to which Fab::antigen binding may influence its organization were analyzed with a specific focus on the ability of this domain to engage immune receptors. Based on the results, an inter-domain allosteric communication was hypothesized between Fab, hinge and Fc upon antigen binding. Previous computational studies have already suggested that antigen binding can induce allosteric effects within Fab domains, suggesting intramolecular communication pathways (44). Systematic analyses further demonstrated that antigen engagement can cause structural rearrangements within the Fab domains, including modifications of CDR loops, VH–VL orientation, and segments of the CH1 domain (4). While these studies focused on local effects, our simulations of full-length mAbs::antigen complexes indicate that these perturbations can extend beyond the Fab, propagating through the hinge and the Fc domain inducing conformational rearrangements. This emerging view aligns with more recent computational evidence. In particular, using classical MD simulations, Zhao et al. (45) suggested that antigen engagement can allosterically promote Fc receptor recognition by altering the conformational ensemble of antibodies.

Building on this foundation, the present work extends this framework showing that antigen binding reshapes not only Fc dynamics but the entire three-dimensional organization of therapeutic mAbs, modulating FcγRIIIa accessibility across glycosylation states and LC isotypes. These findings reveal that Fab engagement plays a central role in regulating global IgG1 architecture. Although the regulation of NK cell-mediated ADCC has been extensively linked to well-established determinants, such as antigen density on the target cell (46, 47) the biophysical properties of the cell membranes (47), and IgG Fc glycosylation, particularly core fucosylation (48), this study introduces another layer of regulation represented by the ability of Fab::antigen engagement to reshape the global conformation of the antibody, thereby modulating Fc accessibility, and makes Fab::antigen recognition an additional determinant of the ADCC response. This concept represents a shift from traditional structure/function models that treat Fab and Fc regions as functionally distinct, highlighting instead a coordinated interdomain communication network with immunological consequences. The findings reported herein are well supported by structural descriptors, such as free energy profiles from aMD simulations, and correlation analyses. Free energy landscapes revealed that antigen engagement stabilizes minimum-energy states corresponding to Y-shaped conformations across all the analyzed complexes, regardless of Fc glycosylation or LC isotype. These structural rearrangements substantially differ from the T-shaped conformations observed in unbound mAbs (16), where only G0 adalimumab adopted an open Y-shaped conformation. This suggests that antigen binding promotes more extended global conformations that facilitate spatial arrangements favorable for FcγRIIIa engagement. These rearrangements involve not only the Fabs orientation but also changes in the internal dynamics of the mAb. Consistently, covariance analysis identified strong long-range correlations between the variable domains of the Fab (VH and VL) and the constant ones in the Fc and hinge. This observation is consistent with previous computational findings on Fab::antigen complexes, which revealed intramolecular communication pathways within Fab domains and antigen-induced changes in elbow angles and VH–CH1 orientations (4) (44),. The presence of such correlations points out the existence of a regulated network of motions capable of mediating allosteric communication within the mAb. In this context, it is interesting to note that afucosylated variants show stronger positive long-range correlations compared to their fucosylated counterparts, suggesting that core fucosylation may limit the propagation of conformational changes across the antibody structure, further reinforcing its already hypothesized role as structural constraint (16, 26, 33). We previously described similar internal cross-domain correlations in our work on Fab glycosylation in cetuximab, further supporting the hypothesis of allosteric communication among domains (49). Interestingly, early studies performed on IgMs suggested that antigen binding to the Fab domains can induce conformational changes in the Fc domain, indicating a potential bidirectional communication along the antibody structure. Structural variations in the Fc domain can therefore affect the conformation of the variable domains, thereby modulating antigen affinity, specificity, and even epitope recognition (50). Through circular polarization of the fluorescence emitted by the Trp residues, Schlessinger et al. observed antigen-induced alterations in the constant regions of both Fabs and Fc. These changes were abolished upon reduction of inter-chain disulfide bonds, highlighting the importance of hinge integrity for long-range signaling inside the antibody (51). Moreover, the engagement of antigen::mAb complexes has been shown to critically increase Fc::FcγRs interactions (52). In the context of anti-TNF mAbs, for example, binding of adalimumab to membrane TNF has been shown to increase its interaction with low-affinity FcγRs, including FcγRIIIa, as well as to potentiate ADCC response. These effects arise from the formation of large mAb::antigen complexes that expose and organize the Fc surface facilitating receptor binding (53). In addition, experimental studies using HDX-MS and HS-AFM on IgG1s demonstrated that Fab regions can directly interact with FcγRIIIa and undergo conformational changes upon antigen engagement (54). Studies on LALA IgG1 variants similarly showed that antigen binding induces Fc changes that modulate interactions with low-affinity FcγRs (55). These studies describe a two-step mechanism in which Fab engagement primes the Fc for receptor binding and promotes the establishment of interactions required for optimal effector function. Our observations align with and extend these findings, suggesting that antigen-induced structural plasticity is a general mechanism governing both specificity and effector activity.

Focusing specifically on the Fc conformational behavior, in line with previous experimental and computational observations, this study shows that fucosylated variants tend to adopt more closed Fc conformations, with reduced distances between the CH2 domains (16, 17, 26, 33), and different energy-minimum structures with respect to G0 forms. Furthermore, a different flexibility of Fc domain was observed between adalimumab and avelumab, indicating a reduced conformational sampling and a more rigid Fc behavior in the latter. This rigidity likely originates from interactions between domains within the Fabs, that are conferred by the LC isotype. Avelumab, with a λ-LC, consistently displays a higher number of stabilizing CH1-CL contacts and more negative electrostatic energies at this interface compared to adalimumab (with a κ-LC), across both glycosylation states (56) (57). Accordingly, Fab motion is limited, constraining the downstream flexibility at the hinge and restraining the propagation of conformational changes toward the Fc. As a result, the structural rearrangements required for efficient FcγRIIIa engagement are partially inhibited. From a functional perspective, a critical outcome of this antigen-induced allosteric effect would be the modulation of Fc accessibility to immune receptors. SASA calculations of key FcγRIIIa-binding residues (41) show that in adalimumab, antigen binding enhances the exposure of these residues, particularly in the G0 form. This trend is less pronounced in avelumab, where differences between glycosylation states are more contained. In our previous computational study, additional residues were identified as involved in FcγRIIIa interaction (26). However, those residues were shown to primarily contribute to the stabilization of the complex once the interaction is established. In contrast, the residues considered here are known to play a role in the initial recognition of the receptor. For this reason, our analysis specifically focused on the latter set of residues, as their accessibility is more likely to be directly modulated by the conformational rearrangements triggered by antigen binding. In addition to structural descriptors, hydration free energy maps of key Fc residues reveal that antigen engagement increases the thermodynamic likelihood of water interactions at these sites, particularly in G0F systems, where antigen binding compensates for the otherwise buried nature of these residues. Notably, G0 adalimumab maintains a relatively stable hydration profile between the unbound and bound states, indicating a pre-exposed receptor binding surface that is further optimized by antigen engagement. This contrasts with G0F adalimumab, which undergoes a reduction in hydration energy upon binding, highlighting that antigen-induced rearrangements are required to accommodate FcγRIIIa. On the other hand, avelumab exhibits less differences in hydration patterns and solvent accessibility between the G0 and G0F states, which is consistent with the reduced conformational flexibility observed across the conformational analyses. Overall, this work suggests that the conformational behavior of Fc is strongly influenced by the antigen binding, the LC isotype and the N-glycosylation pattern, all factors modulating the allosteric communication among mAb domains and therefore the FcγRIIIa engagement.

**Figure 8:**
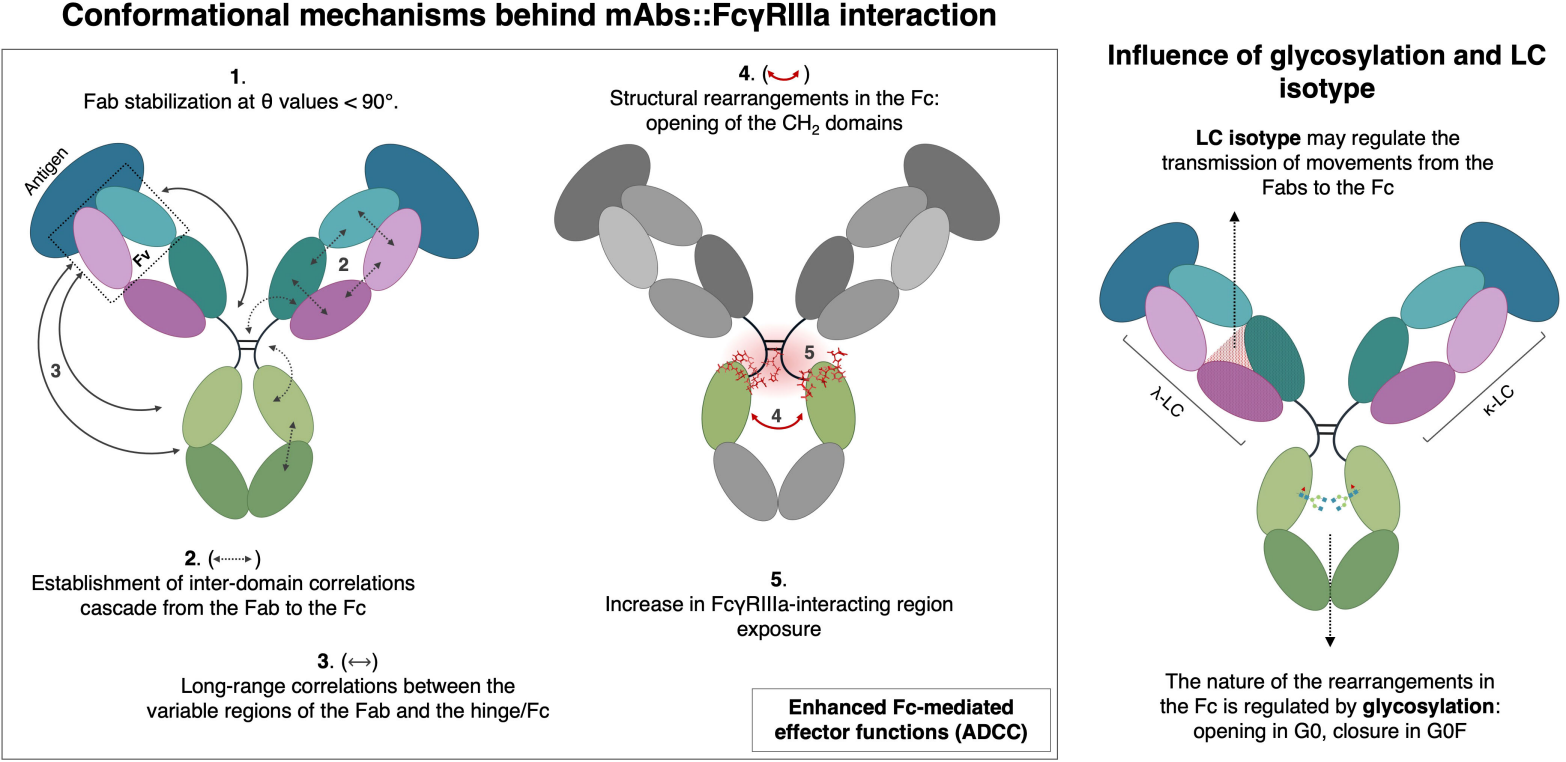
Conformational mechanisms behind mAbs::FcγRIIIa interaction.

To summarize the proposed mechanism, a schematic representation is reported in **Figure 8**, illustrating the cascade of structural events initiated by antigen binding. This visual model highlights the findings that antigen engagement is not functionally limited to the target recognition but actively remodels the antibody structure enhancing Fc-mediated immune effector functions. In this framework, Fab engagement emerges as a critical determinant of global IgG architecture, acting as a structural driver that tunes Fc accessibility and ultimately shapes the effector potential of the mAb. Specifically, the model illustrates how antigen binding promotes long-range interdomain correlations, establishing a dynamic communication network between the variable Fab regions and the hinge/Fc domains. These correlations facilitate structural rearrangements in the Fc, primarily characterized by the opening of the CH2 domains, which ultimately leads to increased exposure of the FcγRIIIa binding interface. Functionally, this structural reorganization results in a greater potential for effector mechanisms such as ADCC. Furthermore, this model incorporates the modulatory influence of both glycosylation and LC isotype. These observations have broad implications for therapeutic mAbs engineering. Effector function optimization has traditionally focused on Fc mutagenesis or glycan remodeling to enhance or decrease FcγRIIIa affinity. The novelty of this study lies in the evidence that the Fab region, through its structure, flexibility, and antigen-induced rearrangement, plays a central role in defining global antibody conformation and therefore Fc-mediated activity, offering a complementary strategy in the optimization of mAbs. By designing mAbs that not only bind antigen with high affinity but also effectively transmit allosteric signals to the Fc region, it may be possible to regulate immune activation in a more integrated and controlled way opening new perspectives in the design of novel biotherapeutics. These computational findings could be further tested and experimentally validated through approaches such as FcγR binding assays and NK cell-mediated ADCC measurements as previously performed by Arora et al. (53), providing a direct link between predicted structural changes and functional outcomes.

## Data Availability

Data will be available on Dataverse, the FAIR research data repository of the University of Milan at the following doi: https://doi.org/10.13130/RD_UNIMI/FULQXI
