# Supplementary material for: Antigen binding triggers long-range conformational changes in monoclonal antibodies
Source: Front Immunol. 2026 Jan 8;16:1680199. doi: 10.3389/fimmu.2025.1680199 (PMC12824876; doi:10.3389/fimmu.2025.1680199)
Supplement: Supplementary file 1 [file SupplementaryFile1.docx]

Supplementary Material

Antigen recognition induces allosteric conformational variability in adalimumab and avelumab

**Davide Bianchi^1^, Simona Saporiti^2*^, Wolf Palinsky^3^, Omar Ben Mariem^1^, Mara Rossi^2^, Ivano Eberini^4,§^ and Fabio Centola^2,§^**

^1^Dipartimento di Scienze Farmacologiche e Biomolecolari, Università degli Studi di Milano, Via Balzaretti, 9, 20133, Milan, Italy

^2^Analytical Excellence and Program Management, Merck Serono S.p.A., Rome, Italy

^3^Global CMC Development, Merck Biopharma, Corsier-sur-Vevey, Switzerland

^4^Dipartimento di Scienze Farmacologiche e Biomolecolari & DSRC, Università degli Studi di Milano, Via Balzaretti, 9, 20133, Milan, Italy

*** Correspondence:**Corresponding Author
[simona.saporiti@merckgroup.com](mailto:simona.saporiti@merckgroup.com)

**§ These authors contributed equally**


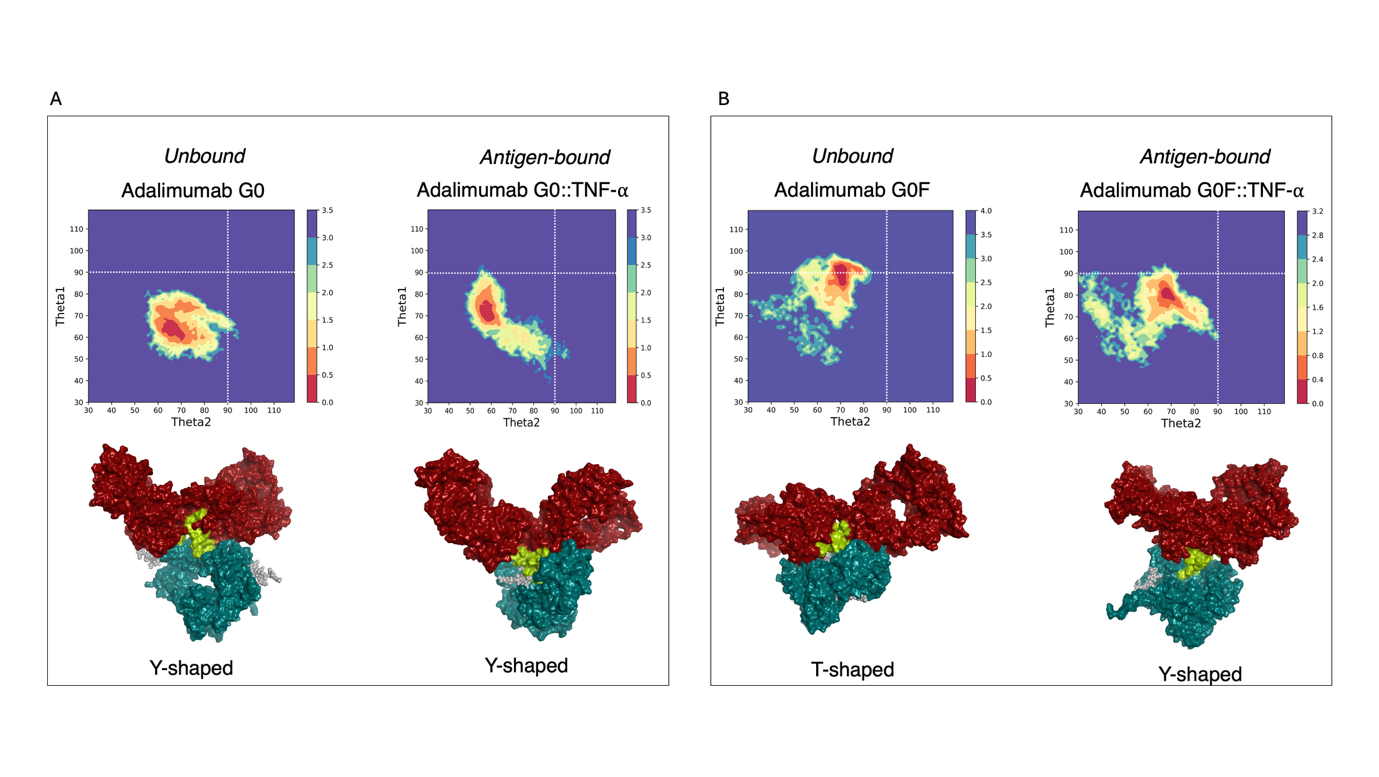


Figure S1. Comparison between the energetic minima identified in the antigen-bound and unbound states in adalimumab G0 (A) and G0F (B).


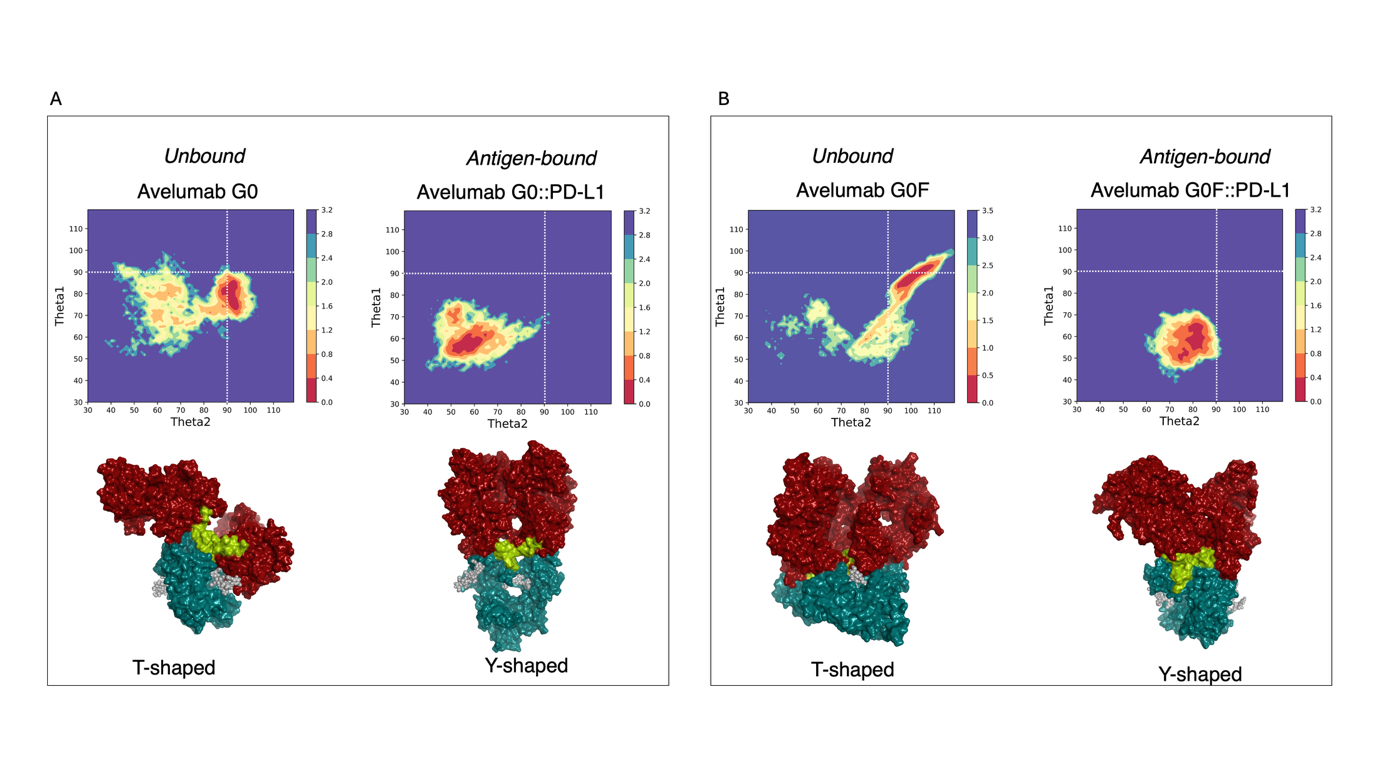


Figure S2. Comparison between the energetic minima identified in the antigen-bound and unbound states in avelumab G0 (A) and G0F (B).


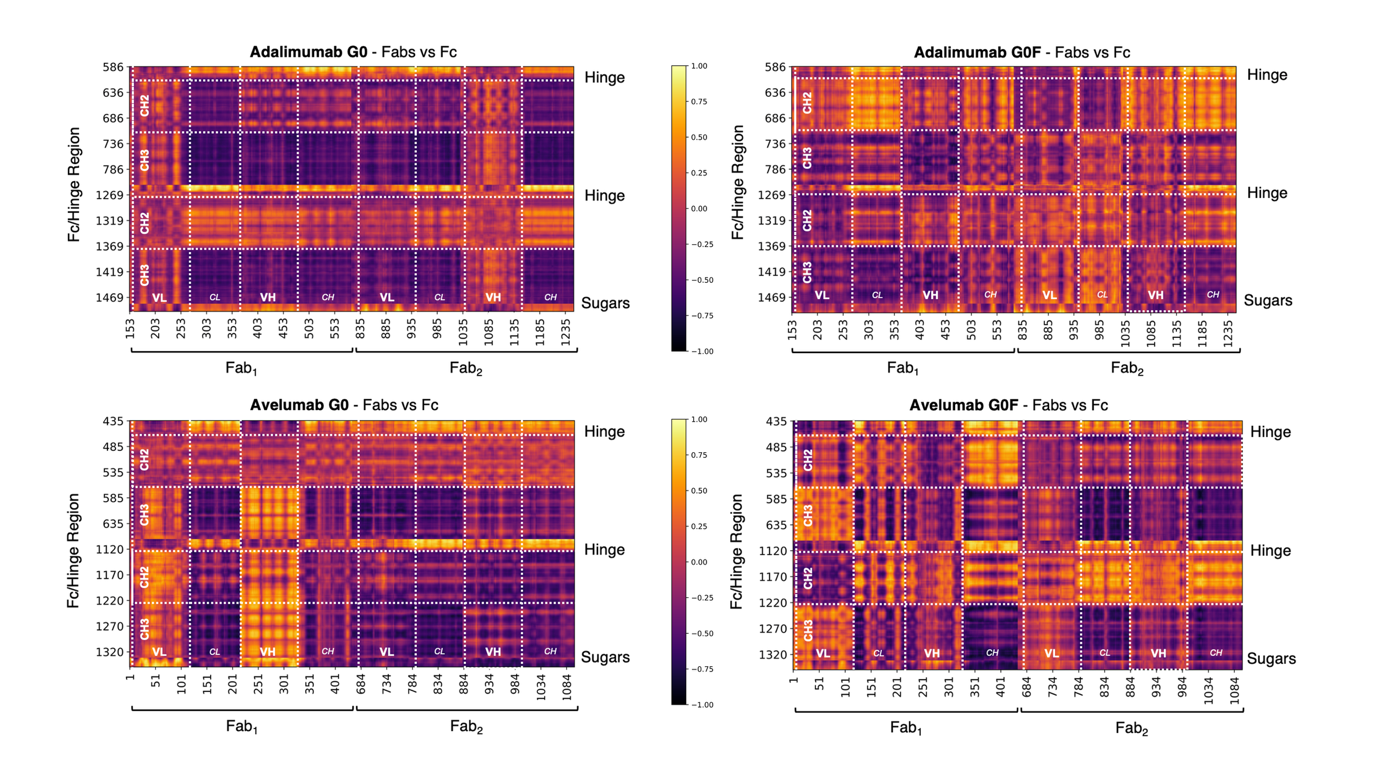


Figure S3. Complete covariance matrices for all mAb::antigen complexes. Yellow: positive correlation; Blue: negative correlations.


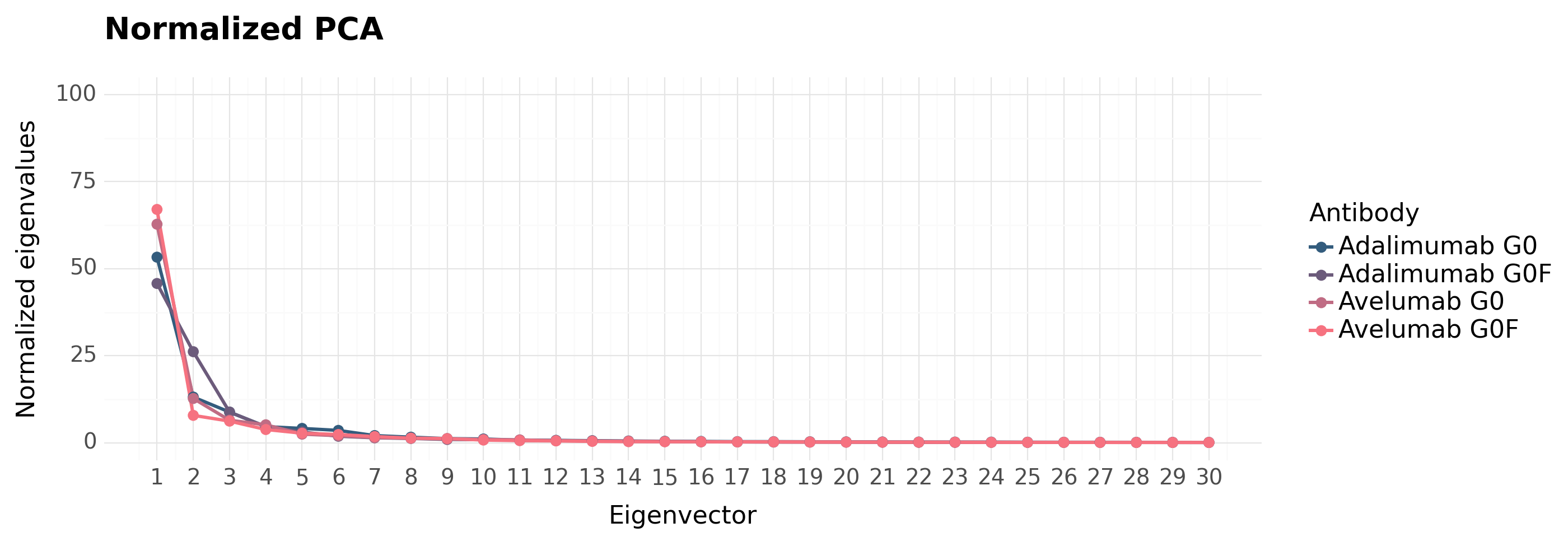


Figure 4. Normalized principal component analysis. The first two principal components describe together more than 65% of the movements exhibited by the antibodies.

Table S1 - EthreshD, EthreshP, alphaD and alphaP values for each mAb::antigen complex.

| Complex | **EthreshD** | **EthreshP** | **alphaD** | **alphaP** |
| --- | --- | --- | --- | --- |
| **G0 adalimumab::TNF-⍺** | 22877 | -3362250 | 1318 | 167933 |
| **G0F adalimumab::TNF-⍺** | 22920 | -3361256 | 1320 | 167895 |
| **G0 avelumab::PD-L1** | 21982 | -2639410 | 1264 | 132544 |
| **G0F avelumab::PD-L1** | 21978 | -2678358 | 1266 | 134457 |
